# Physicochemical Properties and Cell Viability of Shrimp Chitosan Films as Affected by Film Casting Solvents. I-Potential Use as Wound Dressing

**DOI:** 10.3390/ma13215005

**Published:** 2020-11-06

**Authors:** Hugo Yves C. Eulálio, Mariana Vieira, Thiago B. Fideles, Helena Tomás, Suédina M. L. Silva, Carlos A. Peniche, Marcus Vinícius L. Fook

**Affiliations:** 1 Departamento de Engenharia de Materiais, Universidade Federal de Campina Grande, Campina Grande 58429-900, PB, Brazil; hugoyves20@hotmail.com (H.Y.C.E.); suedina.maria@professor.ufcg.edu.br (S.M.L.S.); 2 Centro de Química da Madeira, Universidade da Madeira, Campus da Penteada, 9020-105 Funchal, Portugal; mariana.vieira@staff.uma.pt (M.V.); lenat@uma.pt (H.T.); 3 Coordenação Geral de Inovação Tecnológica na Saúde-CGITS, Departamento de Gestão e Incorporação de Tecnologias e Inovação em Saúde-DGITIS da Secretaria de Ciência, Tecnologia e Insumos Estratégicos do Ministério da Saúde SCTIE/MS, 70058-900 Brasília, Brazil; tfideles@gmail.com; 4 Centro de Biomateriales, Universidad de La Habana, 10400 La Habana, Cuba; peniche@reduniv.edu.cu

**Keywords:** chitosan, film, solubility, organic acids, solvent casting, wound dressing

## Abstract

Chitosan solubility in aqueous organic acids has been widely investigated. However, most of the previous works have been done with plasticized chitosan films and using acetic acid as the film casting solvent. In addition, the properties of these films varied among studies, since they are influenced by different factors such as the chitin source used to produce chitosan, the processing variables involved in the conversion of chitin into chitosan, chitosan properties, types of acids used to dissolve chitosan, types and amounts of plasticizers and the film preparation method. Therefore, this work aimed to prepare chitosan films by the solvent casting method, using chitosan derived from *Litopenaeus vannamei* shrimp shell waste, and five different organic acids (acetic, lactic, maleic, tartaric, and citric acids) without plasticizer, in order to evaluate the effect of organic acid type and chitosan source on physicochemical properties, degradation and cytotoxicity of these chitosan films. The goal was to select the best suited casting solvent to develop wound dressing from shrimp chitosan films. Shrimp chitosan films were analyzed in terms of their qualitative assessment, thickness, water vapor permeability (WVP), water vapor transmission rate (WVTR), wettability, tensile properties, degradation in phosphate buffered saline (PBS) and cytotoxicity towards human fibroblasts using the resazurin reduction method. Regardless of the acid type employed in film preparation, all films were transparent and slightly yellowish, presented homogeneous surfaces, and the thickness was compatible with the epidermis thickness. However, only the ones prepared with maleic acid presented adequate characteristics of WVP, WVTR, wettability, degradability, cytotoxicity and good tensile properties for future application as a wound dressing material. The findings of this study contributed not only to select the best suited casting solvent to develop chitosan films for wound dressing but also to normalize a solubilization protocol for chitosan, derived from *Litopenaeus vannamei* shrimp shell waste, which can be used in the pharmaceutical industry.

## 1. Introduction

Chitosan, composed of glucosamine and N-acetylglucosamine, is a natural polysaccharide obtained from the deacetylation of chitin, which is biocompatible, biodegradable, non-toxic, non-immunogenic and non-carcinogenic, [1,2,3,4,5,6,7,8,9,10,11,12,13]. In addition, it presents healing, hemostatic, antimicrobial, anti-inflammatory, antioxidant, mucoadhesive and analgesic properties [14,15,16,17,18,19,20,21,22], and it also accelerates rapid dermal regeneration and re-epithelization [18,23,24,25]. It can be degraded in vivo by several enzymes, mainly lysozymes, generating non-toxic degradation products such as oligosaccharides, which stimulate deposition of collagen fibrils in the extracellular matrix components [26,27]. Moreover, clinical tests have shown that chitosan-based biomaterials do not result in allergic reactions in the human body after implantation, injection, topical application or ingestion [28]. All of these properties add to its low cost of production and the abundance of raw material (renewable resource) makes chitosan an excellent candidate for the dressing material for wound healing applications, as demonstrated by a large number of publications [29,30,31,32,33,34,35,36,37].

Chitosan-based wound dressings can be processed in different forms, such as hydrogels, membranes, scaffolds, sponges or films [34,38]. Among them, the form of the films has been widely studied [30,31,34,38,39,40,41,42,43,44,45,46] since films can be easily applied to wounds. However, in order to use these chitosan films for the intended application, in addition to their ability to promote wound healing, they should be biocompatible, biodegradable, non-toxic and antimicrobial. Furthermore, they must have a thickness compatible with the epidermis, should be permeable to water vapor to control the moisture and gases in the wound healing process, and should be durable, stress-resistant, flexible and elastic in order to bear the stresses exerted by different parts of the body. Moreover, they should be transparent to indicate the presence of infection and should be easy to apply and remove without incurring any trauma during dressing changes [19,23,47,48,49,50].

Although chitosan films have been extensively studied as a potential material for application as wound dressing, their mechanical, thermal, rheological, morphological, biological, and pharmacological properties are inconsistent among authors. This is because all of these properties are influenced by the source of the chitin used to produce chitosan, by the processing variables involved in the conversion of chitin into chitosan, by the chitosan properties, the types of acids used to dissolve chitosan, the types and amounts of plasticizers and the method of film preparation [51,52,53,54,55,56]. In addition, most of the previous works on chitosan-based wound dressings have been done with plasticized chitosan films and using acetic acid as a solvent. In light of this, this study aimed to prepare chitosan films, by solvent casting method, using chitosan derived from *Litopenaeus vannamei* shrimp shell waste, and five different organic acids (acetic, lactic, maleic, tartaric, and citric acids) without plasticizer, in order to evaluate the effect of organic acid type and chitosan source on the physicochemical properties, degradation and cytotoxicity of these chitosan films. The shrimp chitosan used in this study could be a potential appropriate material for wound dressing applications, not only because of its intrinsic properties but also due to its abundance and low cost of production. The findings of this study may contribute to the selection of the best suited casting solvent to fabricate shrimp chitosan films for wound dressing and to establishing a protocol for shrimp chitosan solubilization from *Litopenaeus vannamei* shrimp shell waste.

## 2. Materials and Methods

### 2.1. Materials

Medium molecular weight shrimp chitosan, derived from *Litopenaeus vannamei* shrimp shell waste, manufactured by Northeastern Biomaterials Evaluation and Development Laboratory-CERTBIO (Campina Grande, Brazil) using the methods established to extract chitin from shrimp shell waste [57] and further processing of chitin into chitosan [58] was used in this work. This chitosan is accredited by the National Institute of Metrology, Quality and Technology (INMETRO) at the International Organization of Standardization (ISO)/International Electrotechnical Commission (IEC) 17025:2017 and it is employed for medical applications.

Five acids were selected to assess their ability to dissolve the shrimp chitosan manufactured by CERTBIO. Acetic acid (AA) 99.8% and lactic acid (LA) 85% (Vetec, Duque de Caxias/Rio de Janeiro, Brazil), maleic acid (MA) 99% (Neon Comercial, São Paulo, Brazil), tartaric acid (TA) 99% and citric acid (CA) (Sigma Aldrich, São Paulo, Brazil). All acids were used in stoichiometric quantity in relation to the amine groups of shrimp chitosan. Phosphate buffered saline (PBS) (Sigma Aldrich, São Paulo, Brazil), human fibroblast cells BJh-TERT (Harvard Medical School, Boston, MA, USA) and Dulbecco’s modified Eagle’s Medium (DMEM) (Gibco, Grand Island, NY, USA) were also employed in this study. All aqueous solutions were prepared using distilled water and all chemicals were analytical grade and used as received.

### 2.2. Characterization of Shrimp Chitosan

The viscosity average molecular weight of the shrimp chitosan was determined from intrinsic viscosity measures carried out on a PSL Rheotek viscosimeter (PSL Rheotek, São Paulo, Brazil). The Mark–Houwink equation was used ([η] = KMa) for assessment of the viscosity average molecular weight, where [η] is the intrinsic viscosity, *M* is the molecular weight, K = 1.38 × 10^−4^ and a = 0.85 at 25 °C [59]. The degree of deacetylation (DD) of the shrimp chitosan was determined by infrared spectroscopy using a Perkin Elmer 400 FTIR spectrometer (Perkin Elmer, Beaconsfield, UK) equipped with attenuated total reflectance (ATR). Thus, the ratio between the hydroxyl band at 3450 cm^−1^ and the amide band at 1655 cm^−1^ was used [60].

### 2.3. Film Preparation

Shrimp chitosan powder was dissolved under magnetic stirring at room temperature by using acetic, lactic, maleic, tartaric, or citric acid water solutions to obtain a polymer concentration of 2.0% (*w*/*v*). Firstly, chitosan (2.0 g) was dispersed in water under stirring and afterwards the minimum amount of the selected acid required for chitosan dissolution, stoichiometrically, was added. Once chitosan was dissolved, water was added to achieve a final volume of 100 mL. The resultant solutions were kept under magnetic stirring at room temperature until pH stabilization. The chitosan solutions pH values were 5.10, 4.00, 2.70, 2.96, and 3.11 for acetic, lactic, maleic, tartaric, or citric acid, respectively. Then, each solution was centrifuged at 3500 rpm for 8 min to remove undissolved particles and air bubbles. Finally, each film-forming solution was cast in the center of a Petri dish (with a diameter of 100 mm) and spread. Films were dried in an oven at 37 °C for 48 h. Dried films were carefully peeled out and stored until used (relative humidity (RH) of 40–60% at 25 °C).

The films were labelled in order to indicate the acid used for preparing the solution: CS-AA (chitosan in acetic acid); CS-LA (chitosan in lactic acid); CS-MA (chitosan in maleic acid); CS-TA (chitosan in tartaric acid) and CS-CA (chitosan in citric acid). The shrimp chitosan powder was labelled as CS.

### 2.4. Thickness

For the measurement of the thickness of the shrimp chitosan films, three samples of each composition conditioned at 25 °C and 40–60% RH were used. Thickness was measured in five random points of each sample using a hand-held micrometer Model MDC-25SB Mitutoyo (from Mitutoyo America Corporation, Houston, TX, USA), with an accuracy of 0.001 mm.

### 2.5. Water Vapor Permeability

The water vapor permeability (WVP) of shrimp chitosan films was determined by gravimetry, using the desiccant method (“A” method). Plastic recipient collectors, with a diameter of 8 cm and a depth of 5 cm, containing about 16.5 g of silica gel were tightly sealed with the chitosan films and then weighed. Afterwards, collectors were placed in a desiccator containing a saturated aqueous KCl solution and kept at 25 °C. The weight gain of the collectors due to moisture absorption by the desiccant from the external atmosphere (kept at controlled environments of 32 °C and 50% RH) was monitored over a 20 day period, with weights recorded at 24 h intervals. WVP (expressed as g m/m^2^ d Pa) of the film was calculated as follows:

(1) WVP=(ΔW×L)/(ΔP×A)

where ΔW is the weight gain of the collector per day (g/d) (i.e., the slope of the linear behavior), L is the film thickness (m), ΔP is the vapor pressure differential across the test film (Pa) and A is the area of exposed film (m^2^). At least 3 replicates were produced from each film type.

The water vapor transmission rate (WVTR) was also determined. It was calculated from the slope of the line resulting from a plot of transmitted mass of water vapor versus time, using Equation (2).

(2) $$\mathrm{WVTR}=\frac{\Delta m}{\left(\Delta t.A\right)}$$

where Δm is the weight gain (g) of film samples per day (g/d); Δt is the elapsed time (h) for the film weight gain and A is the permeation area (m^2^).

### 2.6. Surface Hydrophobicity

Contact angle of water (θ) measurement, by the sessile drop method, upon the surface of the shrimp chitosan films was used to examine the surface hydrophobicity of the films. It was determined with a KRÜSS Drop Shape Analyzer model DSA100 (KRÜSS GmbH, Hamburg, Germany). Thus, a 2 μL-droplet of ultrapure water or PBS was deposited on the film surface with a precision syringe, using a needle with a diameter of 0.75 mm [61]. A video camera was used to record the image of the drop, taken by 5 s, and its profile was numerically solved and fitted to Laplace–Young equation. Nine replicated measurements of θ were obtained.

### 2.7. Tensile Properties

The tensile properties of shrimp chitosan films, namely tensile strength (TS), elongation at break (EB) and Young’s modulus (YM), were measured using an Instron Universal Testing Machine model 6633 (Instron, Norwood, MA, USA), equipped with a 500 N-static load cell. Film samples were cut into strips (50 × 10 mm^2^) and placed in the pneumatic grips of the testing machine. The initial grip separation was set at 50 mm, and the crosshead speed at 5 mm/min [62,63]. In order to calculate the TS, EB and YM values, the load-displacement data collected by the digital acquisition system were converted to stress-strain records. At least eight strips of each film were tested and an average of 5 measurements were reported.

### 2.8. Degradation Assay

The shrimp chitosan films degradation was investigated by monitoring the mass loss of the films, which were cut into equal sizes (1.0 × 1.0 cm^2^), as a function of exposure time, in phosphate buffered saline (PBS) solution. The chitosan films were accurately weighed (Mi), placed in a solution of PBS at pH 7.0, and incubated at 37 °C. Prior to incubation, the PBS solutions prepared with 100 mL of PBS and 900 mL of water were placed in 50 mL tubes in a laminar flow chamber in order to minimize cross-contamination. At regular intervals (1, 2, 4, and 8 weeks), the films were taken out from the PBS solution, rinsed with distilled water, quickly placed on absorbent paper to remove surface water, and weighted (Mt). Equation (3) was applied to determine the percentage of degradation of the films.

(3) $$\mathrm{Mass}\;\mathrm{loss}=\frac{M_{i}-M_{t}}{M_{i}}\times 100\%$$

where M_i_ is the initial weight and M_t_ is the weight after time t. Each biodegradation experiment was repeated three times, and the percentage of biodegradation was expressed as means ± standard deviations.

### 2.9. XRD Analysis

The X-ray scattering (XRD) of chitosan powder and chitosan films was measured at room temperature (22 ± 5 °C.) on a Shimadzu model XRD-7000 (Shimadzu, Tokyo/Kyoto, Japan) diffractometer with Ni-filtered Cu-Kα radiation in the scattering range of 10 to 40° 2θ, with a resolution of 0.02°, at a scanning speed of 1°/min. The analyses were performed by applying 40 kV of voltage and 30 mA of current.

### 2.10. In Vitro Cytotoxicity

Human fibroblast cells BJh-TERT were selected to evaluate the cytotoxicity of the shrimp chitosan films extracts. Fibroblasts were chosen as the initial cell model since they are known to be crucial in the wound healing process [64]. Then, 3ml of each extract from the degradation assay were collected after 14 days. This period was chosen since it is what the literature reports as being the duration of the tissue regeneration process after a light wound or second-degree burn (~15 days) [65]. Five different concentrations of the film extract in PBS were tested: 2%, 4%, 6%, 8% and 10%. PBS solution with no extract was also used as control for each concentration. The PBS solutions were diluted in Dulbecco’s modified Eagle’s Medium (DMEM).

The cytotoxicity was evaluated by determining cell viability using the resazurin reduction method. Cell viability was determined in relation to a positive control (100%) for each concentration, which consisted of BJh-TERT cells grown in DMEM medium with PBS but no added extract. Cells were seeded onto 96-well plates with a cell density of 10,000 cells/well. Extracts with all different concentrations for each sample were added to the wells, in triplicates. Every two days, the preparation (DMEM + extract) was changed, until a final period of seven days for evaluation. At the end of the period, the old solution was changed to a new one, consisting of DMEM with 10% resazurin, followed by incubation for 3 h at 37 °C/5% CO_2_. After this period, 100 μL of the medium was used to measure the fluorescence of resorufin in a Victor^3^ 1420 PerkinElmer microwell plate reader (λ_exc_ = 530 nm; λ_em_ = 590 nm).

### 2.11. Statistical Analysis

To determine the significance of differences among samples, analysis of variance (ANOVA) was used. Minitab^®^ 19 statistical software [66] was employed to perform the analysis and Tukey’s test was utilized for multiple comparisons. All the assays were done at least in triplicate. Significance of differences was defined at *p* < 0.05.

## 3. Results and Discussion

### 3.1. Qualitative Assessment of the Shrimp Chitosan Films

**Figure 1:**
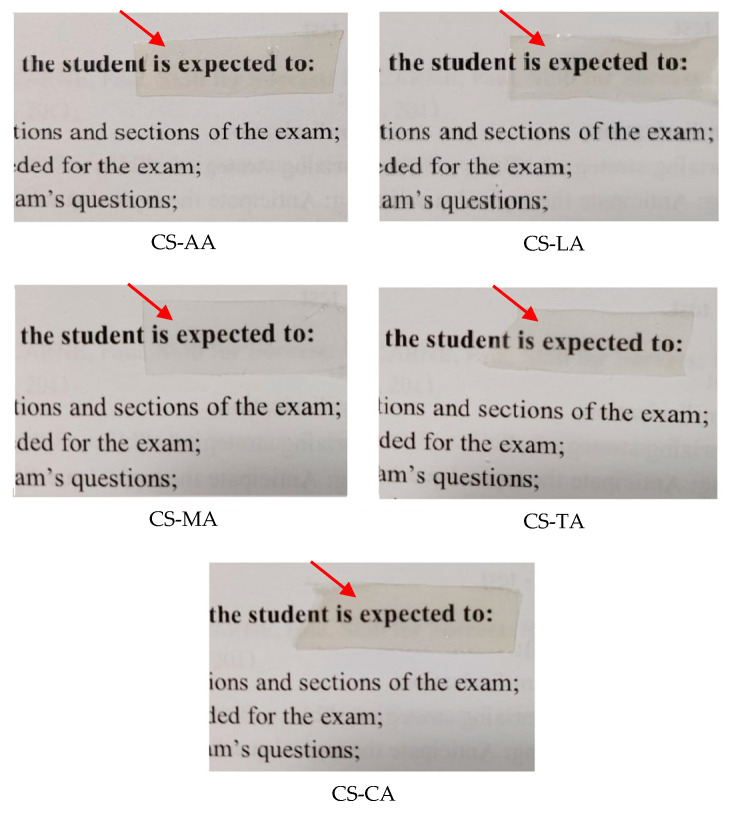
Images of the shrimp chitosan films manufactured with acetic, lactic, maleic, tartaric and citric acids as film casting solvent. A logotype was placed below the samples to better demonstrate the appearance of the films.

In this study, the chitosan extracted from shrimp shell waste (molecular weight 260 kDa and degree of deacetylation 79%) exhibited good film-forming ability. In general, and regardless of the acid type employed in film formation, all shrimp chitosan films were transparent with a slightly yellowish tint as indicated by red arrows in Figure 1. Thus, concerning this physical characteristic, the five acids used to dissolve the shrimp chitosan were shown to be adequate for film manufacturing for wound healing applications, since the wound could be clearly observed, guaranteeing punctual dressing change. Moreover, all shrimp chitosan films were continuous, uniform, and presented homogeneous surfaces without visible pores or cracks. Nevertheless, the hardness, softness and brittleness properties were modified by the acid type used to dissolve the shrimp chitosan. The characteristics of these shrimp chitosan films are described in Table 1.

Although the shrimp chitosan exhibited film-forming ability with all the acids used, only those films formed with acetic, lactic, and maleic acids (CS-AA, CS-LA and CS-MA) were flexible. The ones produced with the tartaric and citric acids (CS-TA and CS-CA) were brittle (Table 1). Brittle CS films would break during handling when used as wound dressing materials. Therefore, according to our results, it is evident that the tartaric and citric acids are not appropriate to produce films using the chitosan from *Litopenaeus vannamei* shrimp shell waste and manufactured by CERTBIO laboratory for the intended applications.

### 3.2. Physicochemical Properties

The results of thickness, water vapor permeability (WVP) and water vapor transmission rate (WVTR) of shrimp chitosan films are presented in Table 2. The thickness of chitosan films was somewhat influenced by the type of film casting solvent used. Shrimp chitosan formed thicker films with citric acid compared to acetic and lactic acids (*p* < 0.05). As for the other solvents used (maleic and tartaric acids), this difference was not significant (*p* > 0.05). Citric acid chitosan-film solutions are known to jellify before the molecules could align and pack [51]; thus, thicker films were obtained with this acid. In addition, due the greater number of hydroxyl and carboxyl groups in the TA and CA acids, possibly all amine groups are protonated in the CS-TA and CS-CA films and thus strong electrostatic interactions around NH_3_^+^ and their counterions can immobilize hydration water. This results in strong rigidity of CS-TA and CS-CA films, preventing the structure from reorganizing, and thus a more amorphous material is obtained, resulting in thicker films. The chain rearrangement in the films, as well as the interactions between the polymer chains, were modified in this case. A more open structure was probably obtained since the presence of the additional carboxyl groups in the citric acid structure can lead to an increase in the intermolecular spaces between the polymer chains, as was previously observed [67,68]. Although all shrimp chitosan films presented thickness compatible with the thickness of the epidermis [69] (from 70 µm to 120 µm in the body surface [70,71]), indicating that they can be considered adequate for wound dressing, due to their brittleness, CS-TA and CS-CA films are not suitable for this application.

The films tested for water transmission determination were CS-AA, CS-LA and CS-MA, since the other two films, CS-TA and CS-CA, were too brittle and fractured during the preparation of the experiment. The WVP and WVTR values of the three films tested are shown in Table 2. The WVP and WVTR values for these films were not significantly influenced by the film casting solvents. It can be deduced that the internal hydrogen bonding of chitosan molecules for the three films are analogous, and thus the intermolecular spacing is equivalent. Therefore, with the free volume and the molecular mobility being identical for the three films, the dissolution of the gas molecules and their transmission through the films was not altered [72]. In addition, the presence of pores in the films, the degree of hydrophilicity and the amount of the polymer crystalline phase must have been similar for CS-AA, CS-LA and CS-MA films and, hence the WVP and WVTR differences were not statistically significant (*p* > 0.05).

The WVTR data obtained here are in the range of those reported by Wiles et al., [56] who found WVTR values ranging from 6.7 to 1146 g/d m^2^ over the range of RH of 11% to 84% for chitosan films that were 39.6 ± 3.6 μm thick. Regarding the values of WVP, Rhim et al. [73] reported a WVP of 1.28 ± 0.01 g·mm/m^2^·h·kPa for a chitosan film measured at 28 to 32 °C. This value is approximately five times higher compared with the one obtained in this study, but this was measured at 95 ± 5% RH. This difference must be influenced by the effect of RH on the WVP of chitosan films. However, as it has been pointed out, care must be taken when comparing WVP values of hydrophilic films, because they are affected by factors such as the measuring method and the experimental conditions (temperature, RH gradient, film thickness, and air gap effect).

Based on the results, shrimp chitosan films produced with acetic, lactic and maleic acids seem to be promising materials for use as a wound dressing. To the best of our knowledge, no clinical data suggest ideal standard values for WVTR that work in practice. However, in the literature studies, the WVTR for the material used for wound healing applications is in the broad range of 76–9360 g/m^2^ day [74,75]. Thus, since the WVTR of the CS-AA, CS-LA and CS-MA films is in range of the ones used for wound dressing applications, we can confirm that these films are appropriate for this use.

### 3.3. Surface Hydrophobicity

**Figure 2:**
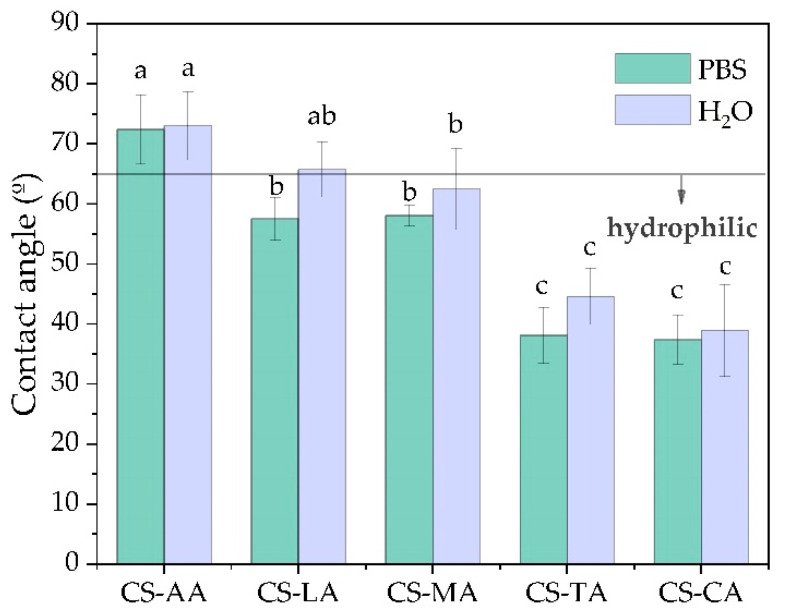
Phosphate buffered saline (PBS) and water contact angle values (average ± standard deviation) for the surface of shrimp chitosan films prepared with acetic, lactic, maleic, tartaric and citric acids. Values labeled with the same letter do not statistically differ from each other (*p* > 0.05).

In dermal applications, wettability is an important property with high impact on the performance of the films. Thus, the surface hydrophobicity of shrimp chitosan films was evaluated by measuring the contact angle of PBS and water (θ) with the film surface by the sessile drop method. The obtained data are shown in Figure 2.

Films with higher θ values present a higher surface hydrophobicity, according to Vogler [76], who states that the quantitative differentiation between hydrophobic and hydrophilic surfaces is based on whether θ > 65° or θ < 65°, respectively. As shown in Figure 2, shrimp chitosan films prepared with acetic acid (CS-AA) can be considered to have hydrophobic surfaces, since θ values are 72.41 ± 5.78° and 73.04 ± 5.69° for PBS and water upon the film surface, respectively. Conversely, all other films, the ones prepared with lactic, maleic, tartaric and citric acids (CS-LA, CS-MA, CS-TA and CS-CA), could be considered to have hydrophilic surfaces, for both PBS and water, since the θ values were below 65° (Figure 2).

It is also apparent in Figure 2 that θ of shrimp chitosan films decreased proportionally to the increase in the hydroxyl and carboxyl group number of the acids. Such behavior can be attributed to the fact that the higher this number is in the acids, the higher the hygroscopic nature, which increases the affinity of the film surface with PBS and water [77]. CS-AA films showed significantly higher values (*p* < 0.05) of θ than those for CS-LA, CS-MA, CS-TA, and CS-CA films. Thus, θ values follow the next order CS-AA > CS-LA and CS-MA > CS-TA and CS-CA for both PBS and water upon the film surfaces. The θ values for CS-LA and CL-MA do not statistically differ from each other (*p* > 0.05), the same was observed for CS-TA and CS-CA films.

The results of the contact angle measurements suggest that the surface hydrophobicity of shrimp chitosan films depends on the acid type employed in the film formation. However, it is evident that even if hydrophilic films have been obtained when shrimp chitosan was solubilized in the tartaric and citric acids, these are not appropriate to produce films using the shrimp chitosan grade manufacture by CERTBIO laboratory for dermal applications, due to their brittleness. Therefore, the most suitable films for the intended application are those prepared with lactic and maleic acids (CS-LA and CS-MA).

### 3.4. Tensile Properties

**Figure 3:**
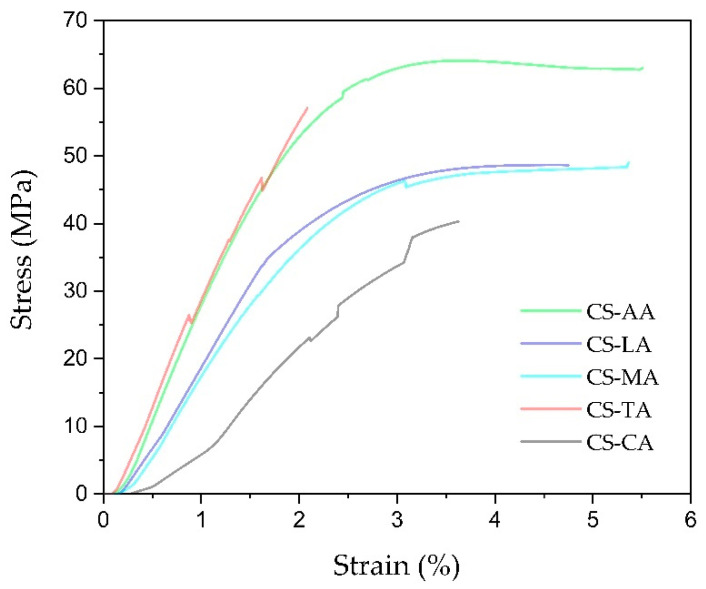
Stress-strain curves of shrimp chitosan films prepared with acetic, lactic, maleic, tartaric and citric acids (CS-AA, CS-LA, CS-MA, CA-TA and CS-CA, respectively).

Representative stress–strain curves of the shrimp chitosan films (CS-AA, CS-LA, CS-MA, CS-TA and CS-CA) are presented in Figure 3, and their tensile strength (TS), elongation at break (EB) and Young’s modulus (YM) are summarized in Table 3.

Results indicate that the type of acid used as film casting solvent influences the tensile properties (TS, EB and YM) of shrimp chitosan films. This is because the type of acid used in preparing the film might affect both the junction density and topological limitations of the film due to the different interaction between chitosan and acid solution, as stated by Kienzle-Sterzer et al. [78]. CS-AA, CS-LA and CS-MA films exhibited plastic deformation, whereas this was not observed for CS-TA and CS-CA films (Figure 3). In addition, more resistant films were obtained when acetic, lactic and maleic acids were used as film casting solvent, as shown by higher TS values (Table 3). It is evident that the cohesive forces in the CS-AA, CS-LA and CS-MA films are more intense than in CS-TA and CS-CA films, suggesting that the former have a tighter structure than the latter, corroborating the thickness data shown in Table 2.

Analyzing the EB data, an indicator of the flexibility and stretchability of the films, it is observed that films prepared with tartaric acid (CS-TA) were the toughest (lowest EB values) and films prepared with lactic acid (CS-LA) were the most resilient (greatest EB values) among the films tested. However, an independent statistical analysis permitted to verify that EB values for CS-AA, CS-LA, CS-MA and CS-CA films were not significantly (*p* > 0.05) different (Table 3). However, the tartaric and citric acids, when used as film casting solvents, did not favor the extension and plastic deformation of the films (Figure 3). Regarding the YM, CS-AA and CS-TA showed significantly higher values (*p* < 0.05) than those for CS-LA, CS-MA, and CS-CA (Table 3). In general, CS-CA films were the weakest with significantly (*p* < 0.05) lower YM compared to the others.

Since the CS-TA and CS-CA films are more hydrophilic than the CS-AA, CS-LA and CS-MA films, as shown by the contact angle data (Figure 2), it seems contradictory to state that they are more brittle because water could plasticize them. However, due to the greater number of hydroxyl and carboxyl groups in TA and CA acids, possibly all amine groups are protonated in CS-TA and CS-CA and thus the water molecules in these films are tightly bound to chitosan and its counterions, immobilizing hydration water. In addition, the possibility of chemical exchange between NH_2_ and NH_3_^+^ groups must be strongly limiting, resulting in less mobility of chitosan chains in CS-TA and CS-CA films than in all the other films. These results are in agreement with previous datum [79] that stated that “the film with the highest moisture content are not the film with the highest mobility”.

When comparing our data to that in the literature, there is much variation among different authors. For instance, Rhim, Weller and Ham [73] reported 41.6, 25.9, 26.7, and 27.3 MPa for TS of chitosan films with acetic, citric, lactic, and maleic acids, respectively. Park et al. [80] reported that TS of chitosan films prepared with acetic acid and maleic acid were 65.96 and 12.72 MPa, respectively. Khan, Peh and Ch’ng [23] reported 67.11 Mpa and 59.87 MP for TS of chitosan films with acetic and lactic acids, respectively. Park, Marsh and Rhim [40] prepared chitosan films using acetic, maleic, lactic and citric acids, and found TS values of 68.8 Mpa, 17.1 Mpa, 27.4 Mpa and 6.7 Mpa, respectively. Altiok et al., [41] reported that TS of chitosan films prepared with acetic acid was 51.2 MPa. Zhang et al. [81] reported that TS of chitosan films prepared with acetic acid was 6.64 MPa. Finally, Taheri et al. [82] reported that TS of chitosan films prepared with acetic acid was 96.77 MPa. In the same way that was observed for TS, comparing our EB and YM data to that in the literature, there is also variation in the observations reported by the same authors that were already cited [23,40,41,73,80,81,82]. Differences in the sources of chitin used to produce chitosan, chitosan properties and methods of film preparation can explain the variation in the tensile properties between authors. Thus, for the chitosan derived from *Litopenaeus vannamei* shrimp shell waste and manufactured by CERTBIO, the organic acids that give better tensile properties to chitosan films were acetic, lactic, and maleic acids.

Overall, all shrimp chitosan films manufactured can be considered adequate for wound dressing application in areas of the body, such as the back and abdomen, since the TS, EB and YM values obtained from our study were higher than those reported by literature for the back and abdomen skin. Karimi and Navidbakhsh [83] achieved TS of 0.95 MPa and 0.78 MPa for the back and abdomen skin, YM of 20.49 MPa and 9.11 MPa for the back and abdomen skin and EB of 3.77% and 4.44% for the back and abdomen skin. The data stated by Karimi and Navidbakhsh is in good agreement with those recorded by Özyazgan et al. [84].

Although all shrimp chitosan films manufactured have adequate tensile properties for wound dressing application, due to their brittleness, the CS-TA and CS-CA films must not be designated for this purpose. In addition, since CS-AA films have hydrophobic surfaces, as previously shown (Figure 2), they are also not acceptable for this application. Thus, only CS-LA and CS-MA films are recommended for wound dressing application in areas of the body, such as the back and abdomen.

### 3.5. Degradation Assay

**Figure 4:**
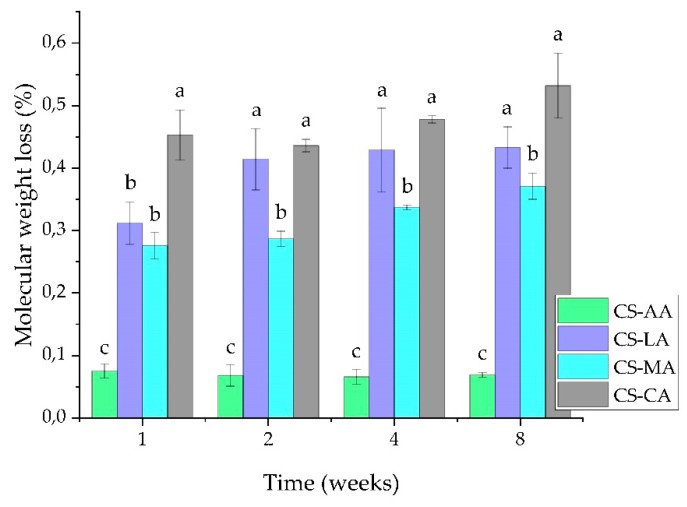
Molecular weight loss percentage in shrimp chitosan films as a function of degradation time. Values labeled with the same letter do not statistically differ from each other (*p* > 0.05).

**Figure 5:**
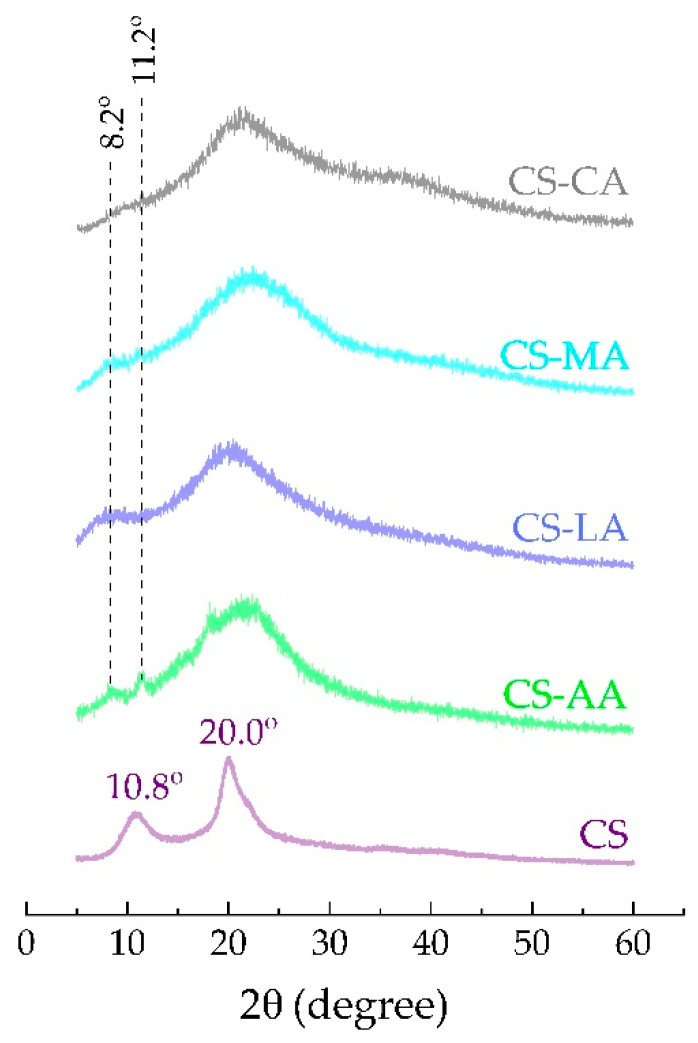
X-ray diffractograms of shrimp chitosan (CS) powder, CS-AA, CS-LA, CS-MA and CS-CA films.

The study of degradation of the shrimp chitosan films was conducted in PBS with pH 7.0 and the results obtained are shown in Figure 4. The films that presented the least degradation in PBS were CS-AA followed by CS-MA and CS-LA films. CS-CA films showed higher degradation values. According to the literature [85], the greater the crystallinity, the lower the degradation rate, which is in accordance with the fact that the CS-AA, CS-MA and CS-LA films showed more crystalline XRD patterns (Figure 5) and lower degradation rates than CS-CA films. Indeed, the type of acid used as film casting solvent was found to degrade chitosan films to varying degrees. Nevertheless, the stability of the films was maintained since the same weight loss percentage was recorded until the end of the eight weeks for all the films prepared in each acid type.

Figure 5 shows the XRD spectra of the raw chitosan powder (CS) and the CS-AA, CS-LA, CS-MA and CS-CA films. The analyses were carried out at room temperature on samples previously air dried for 48 h.

The CS used in this study shows a partially crystalline structure, with two major reflections in 2θ at 10.8° (d-spacing 8.2 Å) and 20.0° (d-spacing 4.4 Å). These peaks are typical fingerprints of chitosan related to hydrated crystals (called type II crystals) and anhydrous crystals (called type I crystals), respectively [13]. This is expected from chitosan with a relatively low degree of acetylation (21%), and it is in agreement with previously reported results [86,87,88].

As can be seen from Figure 5, CS-AA, CS-LA, CS-MA, and CS-CA films are much less organized than CS as indicated by broader reflections at near 20°. In addition, comparing all the curves, their diffraction peaks do not occur at the same position, indicating that the crystal structure in chitosan films is not the same as the crystal structure in CS. This confirms that there was a change in the crystal structure during the chitosan dissolution process. Analyzing each curve, it is possible to observe that (i) CS-AA film gave three reflection angles 2θ = 8.2°, 11.2° and 21.5°. According to literature, peaks at 2θ around 10° (or peaks at ~8° and 12°) are observed in pure hydrated chitosan, partly dried chitosan film immersed in a NaOH solution or hydrated chitosan salts [89,90]. (ii) CS-MA film also presented three reflection angles 2θ = 8.2°, 11.2° and 21.5°, but with less intensity, demonstrating a worse long-range ordering in this film than in CS- [13,89,91,92,93,94]. Lastly (iii) CS-LA film showed only two reflection angles at ~8° and at 20.3°, indicating the presence of a very small amount of hydrated crystal of chitosan and (iv) CS-CA film did not show a reflection angle at ~8° nor at 11.2°, indicating that it is much less organized than the other films. The strong rigidity of CS-CA films may prevent the structure from reorganizing, which is needed to accomplish long-range order [79]. In addition, the diffraction peak that appears in the XRD profile of CS-CA at near 35° can be attributed to the reorganization of CS chains [95]. This peak was observed only for CS-CA because in this case it was not possible to neutralize the density of positive charges on CS, which prevail as NH_3_^+^ due to the greater number of hydroxyl and carboxyl groups in this acid. These results indicate that the type of acid used as film casting solvent influences the crystal lattice of shrimp chitosan films.

### 3.6. In Vitro Cytotoxicity Assay

**Figure 6:**
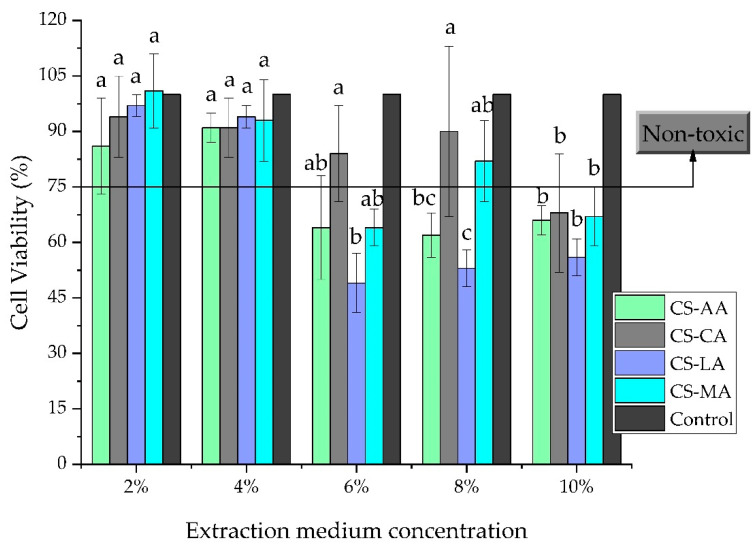
Cytotoxicity of shrimp chitosan films prepared with acetic, citric, lactic, and maleic acids (CS-AA, CS-CA, CS-LA, and CS-MA) in BJh-TERT human fibroblast cells for 7 days after cell seeding at five different concentrations of the film extract in PBS medium (2%, 4%, 6%, 8% and 10%). Same letter indicates that there is no significant difference (*p* > 0.05) by the Tukey’s test.

Shrimp chitosan films prepared with acetic, lactic, maleic, and citric acids were tested for cytotoxicity towards BJh-TERT human fibroblasts cells using the resazurin reduction method. Although other cell lines (for example, human dermal fibroblasts) should also be tested, this cell line was chosen for these first assays since it is known to be involved in the wound healing process [64]. The extracts used in the cell viability assay were the ones obtained in the degradation assay after 14 days, the usual time that the literature reports for the process of tissue regeneration after a light wound or second degree burn [65]. Cells were seeded in DMEM medium with five different concentrations of film extract in PBS medium (2%, 4%, 6%, 8% and 10%) for seven days. The results are expressed as a percentage in relation to the control (cells with no extract). A material is considered cytotoxic when the cell viability is lower than 75%. Taking this into consideration, we could observe in Figure 6 that for 2% and 4% extract concentrations, all shrimp chitosan films were noncytotoxic against BJh-TERT cells. For the 6% concentration, the only non-toxic chitosan films were those prepared with citric, acetic, and maleic acids (CS-CA, CS-AA and CS-MA). For the 8% concentration, only CS-CA and CS-MA were considered as noncytotoxic and for the 10% concentration, all chitosan films were cytotoxic. Noteworthy, the relative cell viability decreased as the concentration of the extract increased. Similar results were reported by Zhao et al., [96] who, using chitosan/glycerophosphate hydrogel with chitosan dissolved in various acids, observed that cell viability increased with decreasing hydrogel extract concentration.

It could be concluded that the degradation products of all shrimp chitosan films prepared were cytocompatible when the extraction medium concentration was less than or equal to 4%. For extraction medium concentrations higher than 4% (but lower than 10%), only those derived from the films made with citric and maleic acids (CS-CA and CS-MA) presented low cytotoxicity. According to the literature [97,98,99], the cytotoxicity of cationic polymers is directly related to their surface charge density. Thus, the higher content of carboxyl groups in the molecule of the citric and maleic acids could be related with the good cellular viability values when they were grown in the presence of the CS-CA and CS-MA extracts at a concentration higher than 4%. Although more studies regarding biocompatibility of the shrimp chitosan films need to be done, these preliminary results that show non-cytotoxicity of their degradation products are promising.

Even though the results indicate that both chitosan films (CS-CA and CS-MA) appear promising as safe biomaterials with potential for wound healing applications, due to their brittleness the CS-CA films cannot be considered for this application. Thus, only CS-MA films could potentially be used for the proposed application. Moreover, it is possible that CS-MA films can accelerate the hemostasis of the wound by coalescing erythrocytes and forming a blood clot, characteristics that were stated in the literature because of the cationic nature of chitosan (Jayakumar et al., 2011).

## 4. Conclusions

Shrimp chitosan films, without plasticizer, were prepared by the solvent casting method using five different organic acids (acetic, lactic, maleic, tartaric, and citric acids) to solubilize chitosan derived from *Litopenaeus vannamei* shrimp shell waste. According to the results, the organic acid type used to solubilize shrimp chitosan influenced the physicochemical properties, degradation and cytotoxicity of the films. Although all shrimp chitosan films prepared with the different acids exhibited film-forming ability, homogeneous surfaces and good transparency in visible light with a slightly yellowish tint, only those manufactured with acetic, lactic, and maleic acids presented flexibility and mechanical resistance when manipulated. All shrimp chitosan films presented thickness compatible with the thickness of the epidermis even though thicker films were obtained with citric acid. WVP and WVTR data of the chitosan films manufactured with acetic, lactic, and maleic acids are in agreement with the ones stated for wound dressing applications. Regarding wettability, chitosan films manufactured with acetic acid had a hydrophobic surface in both distilled water and PBS. Conversely, films prepared with lactic, maleic, tartaric and citric acids had hydrophilic surfaces with the higher wettability presented by those films with tartaric and citric acids. The films prepared with acetic, lactic and maleic acids exhibited plastic deformation, whereas this did not occur for the films prepared with tartaric and citric acids. In addition, more resistant films were obtained when acetic, lactic and maleic acids were used as film casting solvents. The type of acid used was found to degrade chitosan films to varying degrees. The films that presented the least degradation in PBS were the ones prepared with acetic and maleic acids. The films prepared with citric and maleic acids also had appropriate cytocompatibility and appear promising as safe biomaterials with potential for wound dressing applications. However, due to its brittleness, one can conclude that films prepared with citric acid are not suitable for this application. As a result, and according to the physicochemical properties and cytotoxicity of the films, the only acid potentially adequate for manufacturing shrimp chitosan films to be used in the pharmaceutical industry as a dressing material for wound healing applications is maleic acid. The outcome of this study contributes not only to a better understanding of the effect of the organic acid type used as a casting solvent in shrimp chitosan films on its physicochemical properties, degradation and cytotoxicity, and also for the establishment of a protocol for chitosan solubilization from *Litopenaeus vannamei* shrimp shell waste. However, more studies are needed to confirm the usefulness of this maleic acid-based shrimp chitosan film as a reliable and safe material in wound dressings, such as films biocompatibility, wound healing assay, permeability to oxygen, adhesive nature, antifungal and bactericidal character, as well as preclinical and clinical studies.

## Figures and Tables

**Table 1:** Characteristics of shrimp chitosan films formulated with different organic acids.

Film Sample	Film Properties
CS-AA	A transparent, slightly yellowish, moderately flexible, non-sticky film with smooth soft surface and acidic odor
CS-LA	A transparent, slightly yellowish, flexible, non-sticky film with smooth, very soft surface and odorless
CS-MA	A transparent, slightly yellowish, moderately flexible, non-sticky film with smooth, very soft surface and odorless
CS-TA	A transparent, slightly yellowish, brittle, non-sticky film with slightly rough surface and odorless
CS-CA	A transparent, slightly yellowish, brittle, non-sticky film with smooth surface and odorless

**Table 2:** Physicochemical properties of shrimp chitosan films.

Film Sample	Thickness (µm)	WVP (g m/m^2^ d Pa)	WVTR (g/m^2^.h)
CS-AA	78.80 ± 12.50 ^b^	5.10 ± 0.50 ^a^	8.06 ± 0.48 ^a^
CS-LA	84.60 ± 4.83 ^b^	3.27 ± 0.76 ^a^	8.24 ± 0.78 ^a^
CS-MA	93.60 ± 18.76 ^ab^	4.00 ± 0.40 ^a^	7.24 ± 0.33 ^a^
CS-TA	100.60 ± 12.72 ^ab^	-	-
CS-CA	112.60 ± 15.98 ^a^	-	-

^a,b^ Different superscript lowercase letters within the same row indicate significant differences by the Tukey test (*p* < 0.05).

**Table 3:** Tensile properties of shrimp chitosan films prepared with acetic, lactic, maleic, tartaric and citric acids (CS-AA, CS-LA, CS-MA, CA-TA and CS-CA, respectively).

Film Sample	TS (MPa)	EB (%)	YM (MPa)
CS-AA	62.03 ± 4.93 ^a^	3.19 ± 0.65 ^ab^	33.63 ± 1.59 ^a^
CS-LA	50.70 ± 3.74 ^ab^	4.10 ± 2.34 ^a^	24.78 ± 1.03 ^b^
CS-MA	44.71 ± 8.83 ^bc^	3.86 ± 1.65 ^ab^	23.55 ± 1.12 ^b^
CS-TA	36.76 ± 9.11 ^cd^	1.34 ± 0.51 ^b^	35.79 ± 0.76 ^a^
CS-CA	25.24 ± 2.47 ^d^	2.79 ± 0.59 ^ab^	16.03 ± 2.49 ^c^

^a–d^ Different superscript lowercase letters within the same row indicate significant differences by the Tukey test (*p* < 0.05).
